# Neural Networks for the Detection of COVID-19 and Other Diseases: Prospects and Challenges

**DOI:** 10.3390/bioengineering10070850

**Published:** 2023-07-18

**Authors:** Muhammad Azeem, Shumaila Javaid, Ruhul Amin Khalil, Hamza Fahim, Turke Althobaiti, Nasser Alsharif, Nasir Saeed

**Affiliations:** 1 School of Science, Engineering & Environment, University of Salford, Manchester M5 4WT, UK; azeemchaudharyg@gmail.com; 2 Department of Control Science and Engineering, College of Electronics and Information Engineering, Tongji University, Shanghai 201804, China; shumaila@tongji.edu.cn (S.J.); hamzafahim@tongji.edu.cn (H.F.); 3 Department of Electrical Engineering, University of Engineering and Technology, Peshawar 25120, Pakistan; ruhulamin@uetpeshawar.edu.pk; 4 Department of Electrical and Communication Engineering, United Arab Emirates University (UAEU), Al-Ain 15551, United Arab Emirates; 5 Department of Computer Science, Faculty of Science, Northern Border University, Arar 73222, Saudi Arabia; turke.althobaiti@nbu.edu.sa; 6 Department of Administrative and Financial Sciences, Ranyah University Collage, Taif University, P.O. Box 11099, Taif 21944, Saudi Arabia; n.alsharif@tu.edu.sa

**Keywords:** artificial neural networks, convolutional neural networks, healthcare, infectious diseases

## Abstract

Artificial neural networks (ANNs) ability to learn, correct errors, and transform a large amount of raw data into beneficial medical decisions for treatment and care has increased in popularity for enhanced patient safety and quality of care. Therefore, this paper reviews the critical role of ANNs in providing valuable insights for patients’ healthcare decisions and efficient disease diagnosis. We study different types of ANNs in the existing literature that advance ANNs’ adaptation for complex applications. Specifically, we investigate ANNs’ advances for predicting viral, cancer, skin, and COVID-19 diseases. Furthermore, we propose a deep convolutional neural network (CNN) model called ConXNet, based on chest radiography images, to improve the detection accuracy of COVID-19 disease. ConXNet is trained and tested using a chest radiography image dataset obtained from Kaggle, achieving more than 97% accuracy and 98% precision, which is better than other existing state-of-the-art models, such as DeTraC, U-Net, COVID MTNet, and COVID-Net, having 93.1%, 94.10%, 84.76%, and 90% accuracy and 94%, 95%, 85%, and 92% precision, respectively. The results show that the ConXNet model performed significantly well for a relatively large dataset compared with the aforementioned models. Moreover, the ConXNet model reduces the time complexity by using dropout layers and batch normalization techniques. Finally, we highlight future research directions and challenges, such as the complexity of the algorithms, insufficient available data, privacy and security, and integration of biosensing with ANNs. These research directions require considerable attention for improving the scope of ANNs for medical diagnostic and treatment applications.

## 1. Introduction

Artificial intelligence (AI) is revolutionizing the world with its endless applications. The platforms built on AI are prevalent, accelerating the pace of developing life-saving drugs and reducing operations costs. Artificial neural networks (ANN) are the building blocks of AI technologies, which simulate the human brain’s analyzing and processing abilities to solve complex problems. The unique characteristics of ANN (such as efficient data handling, low complexity, reduced computation, and storage requirements) have enormous potential for a wide range of disciplines, including medical sciences [1] (especially in the areas of cardiology [2], radiology [3], oncology [4], urology [5]), veterinary [6], stock exchange [7], law enforcement department [8], ecology [9], human resource management [10], signal processing with blind separation [11], and cybersecurity [12]. The ANNs are mainly based on mathematical models inspired by biological nervous systems, such as the brain’s route information. They work similarly to an adaptive system, which updates its configuration in the learning phase and can be modelled for a specific application, such as data classification and pattern categorization. A neural network generally consists of three layers (i.e., input layer, hidden layer, and output layer). Based on these layers, ANN can be categorized into a single-layer feed-forward neural network (FFNN) [13], a multilayer feed-forward network [14], a single node with its feedback, and a recurrent multilayer network [15].

The massive potential of various types of ANN models in the healthcare domain must be addressed. Recent literature [16,17] has extensively studied the potential of ANN for the treatment and diagnosis of a wide range of infectious diseases, such as diarrhea [18], tuberculosis [19], dengue [20], COVID-19 [21,22], and childhood blindness [23]. State-of-the-art schemes generally considered accuracy and complexity as the primary performance metrics to examine the performance of ANN.

The development of ANNs has revolutionized disease detection and diagnosis, offering numerous benefits over traditional approaches, having the capability to analyze large volumes of data, extract meaningful patterns, and provide accurate predictions. In the context of COVID-19, the rapid and precise identification of infected individuals is crucial for effective control and mitigation. In particular, those applied to radiography images can play a vital role in automating the analysis process and reducing the workload of healthcare professionals.

Therefore, in this paper, we have proposed a ConXNet model that can effectively classify COVID-19 cases from typical cases, providing timely and reliable support for diagnosis with the potential to transform healthcare practices, enabling efficient online monitoring and enhancing the overall quality of patient care. By highlighting the role of the ConXNet model in disease detection and emphasizing the specific application to COVID-19, our research contributes to the ongoing efforts in combating the pandemic and improving healthcare outcomes. The main contributions of our presented review are summarized as follows.

- First, we present a critical review of state-of-the-art ANN models that have contributed to the detection and diagnosis of various diseases, including skin diseases, retinal diseases, and COVID-19. While numerous studies are available on using ANNs in the medical field, a comprehensive review of these techniques is crucial to understand their current and future potential clearly. Therefore, this paper aims to offer a comprehensive and concise overview of recent advancements in ANNs for medical applications.
- Second, our work focuses explicitly on detecting and diagnosing COVID-19 using convolutional neural network (CNN) models. We provide an in-depth analysis of existing CNN models designed for COVID-19 detection, discussing their contributions and limitations.
- Then, we propose a novel deep learning model called ConXNet, which has been trained and tested using different datasets to improve the accuracy of COVID-19 detection by up to 98%. This contribution is significant as it offers an innovative approach to enhance the detection capabilities of AI-based models in the context of a global health crisis.
- Finally, we highlight the gaps that require attention in the future to improve ANN-based disease diagnosis and treatment. These future research challenges include algorithm complexity, inadequate available data, security and privacy concerns, and biosensing integration with ANNs.

The rest of the paper is organized as follows: Section 2 thoroughly discusses the literature background. The background of ANN is discussed in Section 3. Section 4 provides a comprehensive study of existing CNN models designed for COVID-19. Section 5 describes our proposed ConXNet model, including the details of datasets and obtained results for COVID-19 detection. Section 6 reviews existing CNN models for various diseases. Section 7 provides the Discussion, Opportunities, and Open Issues; Section 8 highlights challenges and future research directions. Finally, Section 9 summarizes the findings of the presented reviews and provides future work directions.

## 2. Background and Related work

In recent years, numerous studies have focused on applying artificial intelligence (AI) techniques for combating the COVID-19 pandemic. In [24], the authors shed light on the opportunities, challenges, and prospects of explainable AI in fighting the pandemic. Their work emphasizes the potential of explainable AI techniques and how they can contribute to addressing the challenges posed by the pandemic. Similarly, [25] presents a scoping review of the challenges and opportunities of deep learning for cough-based COVID-19 diagnosis. By examining existing literature on deep learning approaches for diagnosing COVID-19 through cough analysis, they identify the challenges and discuss the potential of these techniques in improving diagnostic capabilities.

Researchers have explored various deep learning approaches for COVID-19 detection as AI evolves. In [26], the authors surveyed the detection of COVID-19 using deep learning techniques, providing insights into the different approaches employed and evaluating their cost-effectiveness. Furthermore, [27] presents a survey focusing on deep convolutional neural networks (CNNs) for detecting COVID-19 using medical images. Its review highlights the state-of-the-art deep learning models for COVID-19 detection through medical imaging, discussing their strengths, limitations, and potential applications in clinical practice.

For example, the authors in [28] investigated the effectiveness of ANN in detecting skin cancer. Their findings highlighted the exceptional performance of various neural network models, specifically the VGG-16 convolutional neural network (CNN). Notably, CNN achieved an accuracy of 87.6%. Moreover, the ability of advanced convolutional neural networks to distinguish between serious and benign skin cancers is examined in this study. In [29]; the authors performed an extensive study on neural networks for lung cancer detection and concluded that neural network techniques exhibit an excellent classification rate. However, they often require a significant amount of training time. In another work in [30], the authors studied a convolutional neural network, followed by a Pix2pix generative adversarial network (GAN) proposed for image enhancement, and the model successfully detected breast cancer with an accuracy of 78.52%. Although the limitations on the enormous volumes of annotated data may be reduced or even eliminated with the help of GANs, there are still some difficulties, such as the need for a more accurate feature extraction, which may have a significant impact on the practical implementation of ANN-based image processing techniques in digital pathology.

Furthermore, in [31], the authors focused on using convolutional neural networks (CNNs) for detecting malaria infection, a widely spread parasitic infectious disease. The importance of early detection in combating malaria was emphasized due to the limitations and errors associated with current techniques, such as manual microscopic examination and rapid diagnostic tests. Based on a custom CNN with three convolutional layers, the proposed model offers a faster and cost-effective method for distinguishing between healthy and infected blood samples, thus improving parasite (Plasmodium) detection. The proposed model achieved an accuracy of 96.71%. Similarly, in [32], the authors provided a comprehensive review of surveys and recent techniques in brain tumour classification, including preprocessing, feature extraction, and classification steps. The use of convolutional neural network models, transfer learning, and data augmentation, along with their achievements and limitations, is explored. The overview also highlights the importance of personalized and smart healthcare and suggests future research directions for improved brain tumor classification.

Moreover, in [33], the authors discussed various ML algorithms, such as naïve Bayes, classification and regression tree (CART), decision tree (DT), and support vector machine (SVM), for different diseases, such as lung cancer, breast cancer, and skin diseases. In [34], the authors present a comparative study on data mining techniques for breast cancer prediction, including naïve Bayes, back-propagated neural networks, and decision tree algorithms. In [35], the authors investigated the effectiveness of numerous nature-inspired computing techniques, such as genetic algorithms [36], ant colony optimization [37], particle swarm optimization [38], and artificial bee colony [39], for diagnosing various critical human disorders. They concluded that nature-inspired computing techniques have high accuracy for disease detection and diagnosis. However, the survey lacks a detailed comparison with state-of-the-art schemes required to investigate the efficiency of nature-inspired techniques for real-world problems. Similarly, in [40], the authors extensively discuss hybrid models(i.e., neural networks combined with other classical methods or metaheuristic approaches) to outline selecting a well-suited ANN model for epidemic forecasts. Reference [41] shows that hybrid neural networks depict enhanced performance for epidemic forecasts.

Besides other infectious disease detections, recently, ANNs have been explored for COVID-19 detection. For instance, in [42], the authors highlight the role of AI, particularly in diagnosing and treating COVID-19. Moreover, it highlights that disease detection and diagnosis, virological research, drug and vaccine development, epidemic and transmission prediction, medical image analysis, and drug discovery are the primary areas that integrate AI to fight against COVID-19. Furthermore, the authors in [43] illustrated computer vision’s role in combating the COVID-19 pandemic. They considered three types of visionary images for COVID-19 detection: computed tomography (CT) scans, X-ray imagery, and ultrasound imaging.

In addition to image-based diagnostics, researchers have investigated other modalities for COVID-19 detection. In [44], the authors propose a hybrid deep-fused learning approach to segregate infectious diseases, including COVID-19. Their study explores the combination of multiple data modalities to improve the accuracy of disease segregation. On the other hand, in [45], the authors introduce Epi-DNNs, which are deep neural networks informed by epidemiological priors. By integrating epidemiological priors into the neural network models, they aim to enhance the modelling of COVID-19 dynamics and improve forecasting capabilities.

Furthermore, the utilization of non-image-based approaches has also gained attention. In [46], the authors proposes a deep transfer learning-based CNN model for COVID-19 detection using computed tomography (CT) scan images. Their research focuses on improving the accuracy of COVID-19 detection through the application of transfer learning techniques in CT image analysis. Additionally, in [47], the authors investigated the diagnosis of COVID-19 from blood parameters using a CNN. Their study explores the potential of CNN models to analyze blood parameters and detect COVID-19, contributing to the development of non-image-based diagnostic approaches.

Our proposed work makes significant contributions in the context of this existing literature. We comprehensively review artificial neural network (ANN) models applied in medical domains, explicitly focusing on CNN models for COVID-19 detection. We propose a novel CNN model called ConXNet, which outperforms existing models in accuracy. Our work aims to bridge the gap between theory and practice, addressing the challenges and opportunities in COVID-19 detection. By incorporating insights from the literature and developing an innovative model, our research contributes to the ongoing efforts to combat the pandemic. It provides valuable insights for AI-driven disease detection and diagnosis.

## 3. Review of Artificial Neural Networks (ANNs)

Recently, neural networks have gained particular importance due to their diverse applications. Research and scientific communities are optimistic regarding the potential of ANN. The core property of an ANN is its capability of learning. There are three types of learning: (1) Supervised learning, which is accomplished in the existence of an observer. In this type of learning, a supervisor or observer is compulsory for error minimization. (2) Unsupervised learning is accomplished without an observer’s help, and the network itself discovers features, categories, patterns, or symmetries from the input data and associations for the input data over the output. (3) Reinforcement learning is used to train machine learning models. It enables an agent to learn through the consequences of actions in a specific environment.

Hence, many neural networks are developed and categorized over time based on their learning characteristics. In the following, we extensively discuss different types of neural networks to comprehend the background of ANN.

### 3.1. Perceptron

The perceptron model, proposed by Minsky and Papert in the 1950s, is one of the modest and ancient models of the neuron. Perceptron is the most minor component of a neural network that performs particular calculations to identify features in the input data [48]. The perceptron is a supervised learning algorithm that can easily classify the data into two categories by a hyperplane; therefore, it is also called a binary classifier. The perceptron algorithm has gained significant attention recently due to its use in establishing logic gates, such as AND, OR, or NAND.

**Figure 1:**
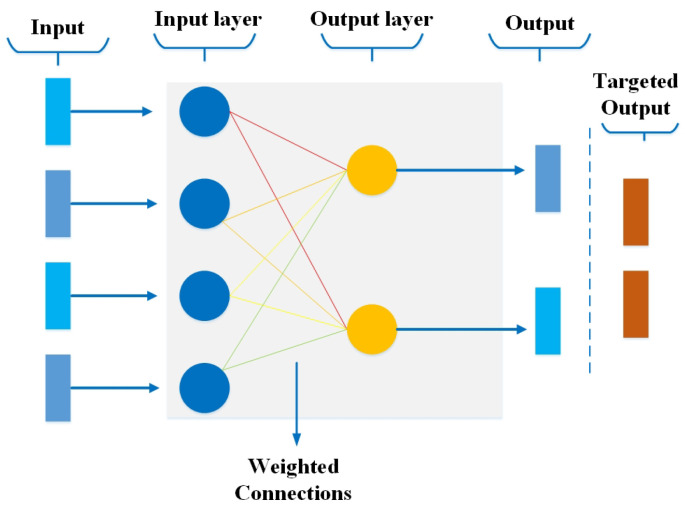
Perceptron architecture.

Figure 1 shows a simple perceptron as an example with a layer of input nodes (layer 1) and a layer of output nodes (layer 2). Every perceptron layer is fully connected, but no links occur among nodes in the same layer. When layer 1 directs a signal to layer 2, the related weights on the links are applied, and each acceptance node on layer 2 sums up the received values. If the sum surpasses a given threshold, that node, in turn, directs an output signal.

The outputs are summed through all the inputs (a[i]) received by a node (*j*) in the output layer. The weighted sum is then combined with an activation function (*f*) to provide an output that can be either binary or continuous. Moreover, this weighted sum can be improved by including a bias value denoted as ‘*b*’. Then, the output of each node is given by

(1) $$S_{j}=f\left(\sum_{i=0}^{n}a_{i}w_{ij}+b\right).$$

in (1), if $S_{j}>\theta$, then $x_{j}=1$; otherwise, $x_{j}=0$, where θ is the threshold. The perceptron’s output can be "trained" to match the desired output by adjusting the weights on the connections between layers. The amount of the correction is determined by multiplying the difference between the actual output (x[j]) and target (t[j]) values by a learning rate constant (*C*). If the nodes’ output (a[i]) is 1, that connection weight is adjusted, and if it sends 0, it has no bearing on the output, and subsequently, there is no need for adjustment. Thus, the process weight adjustment is as follows:

(2) $$w_{new}^{ij}=w_{old}^{ij}+C\left(t_{j}-x_{j}\right)a_{i},$$

where *C* is the learning rate. This training procedure is repeated until the network performance reaches a maximum threshold [49].

### 3.2. Multilayer Perceptron

**Figure 2:**
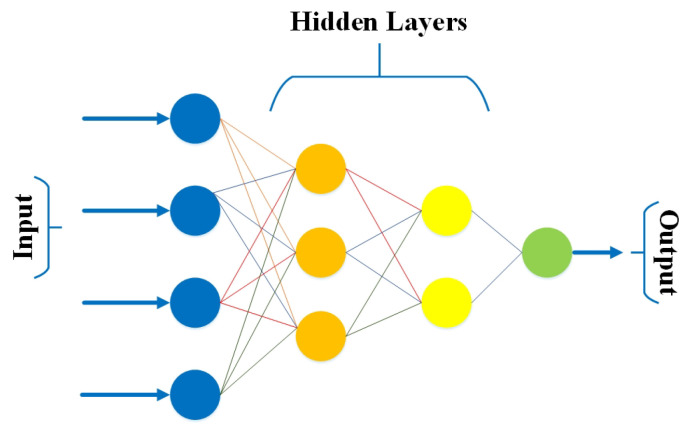
A simple presentation of MLP.

In MLP, every node is associated with all neurons in the subsequent layer, making it a fully connected neural network. Input and output layers exist with many hidden layers (i.e., at least three or more layers) with bidirectional propagation (i.e., forward and backwards). In forward propagation, inputs are multiplied with weights and sent to the activation function, and in backpropagation, the weights are adjusted to reduce the loss [50]. Figure 2 shows a simple presentation of MLP, consisting of an input layer, a hidden layer, and an output layer. Except for the input nodes, each node is a neuron that uses a nonlinear activation function.

### 3.3. Feed-Forward Neural Network (FFNN)

**Figure 3:**
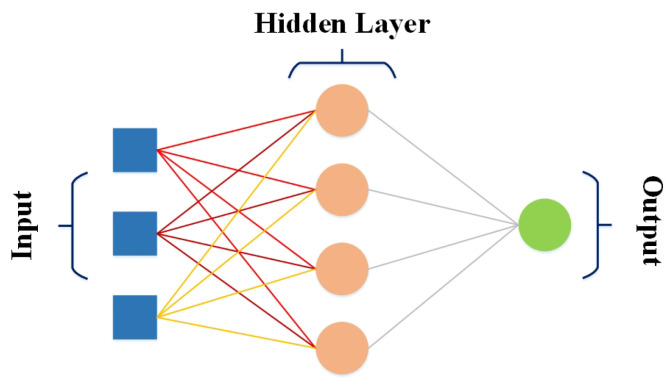
FFNN architecture.

FFNN [51] is the most basic neural network used for different applications, including computer vision, natural language processing, and speech recognition. In FFNN, data propagate only in one direction, passing through neural nodes and exiting through output nodes. FFNN comprises input and output layers; hidden layers may exist. The weights are static in FFNN, and an activation function is used by inputs multiplied by weights to learn the value of parameter θ. In addition, the design of FFNN requires considering several crucial factors, including an optimizer, cost function, and output units. FFNN in Figure 3 shows an input passed through one or more hidden layers unidirectionally, and weights are multiplied by inputs to minimize the error using an optimizer.

### 3.4. Convolutional Neural Network (CNN)

**Figure 4:**
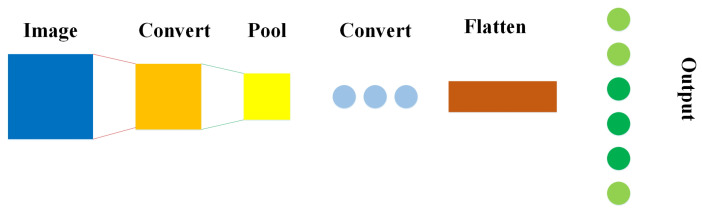
A simple presentation of CNN.

Initially, CNN was only restricted to image processing, requiring large datasets. However, the success of AlexNet in the 2012 ImageNet challenge showed that the time has come to revisit CNNs, as large datasets available [52,53]. A CNN comprises a three-dimensional set of neurons instead of the typical two-dimensional array. The first layer is called a convolutional layer, where every neuron only routes the information from a minor part of the layer. The convolutional layer uses ReLU as an activation function, followed by softmax. The first layer is followed by a pooling layer, where the convolution layer’s output drives to a fully connected neural network for classification. The CNNs show promising results in image and video recognition, semantic parsing, and paraphrase detection. Figure 4 visualizes a CNN model that first extracts all the features of the input image using the convolution operation and then uses the pooling layer to extract the most prominent features to pass through to the other convolutional layer for linearity. Later, the flattened layer shapes the input data for producing an output.

### 3.5. Radial Basis Function Neural Networks

**Figure 5:**
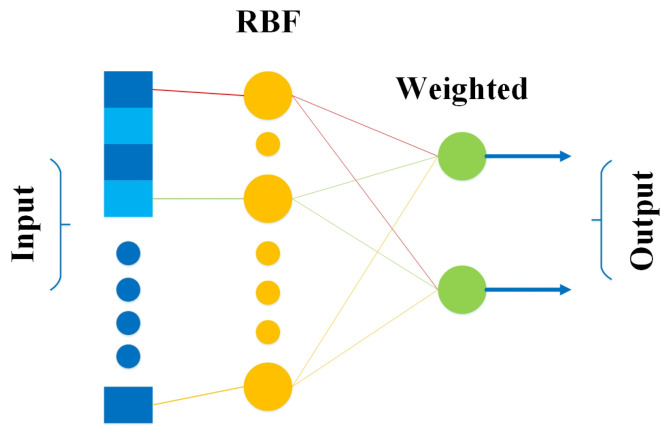
Architecture of RBF.

The radial basis function (RBF) network comprises an input vector, followed by a layer of RBF neurons and an output layer with one node per class. Classification is accomplished by assessing the input’s resemblance to data points from the training set, where each neuron stores a prototype, one of the samples from the training set. When a novel input vector (the n-dimensional vector that you are trying to classify) needs to be organised, every neuron computes the Euclidean distance between the input and its prototype [54,55]. Figure 5 shows the simple architecture of RBF comprising an input layer, a hidden layer (consisting of several RBF nonlinear activation units), and an output layer.

### 3.6. Recurrent Neural Networks (RNNs)

RNN is one of the most potent and robust state-of-the-art algorithms derived from FFNN for modelling sequence data [56]. RNN differs from other algorithms due to its internal memory and ability to process the sequence data lacking in different algorithms. Furthermore, RNN trains the model based on the current input and previous learning experience, offering more precise predictions.

**Figure 6:**
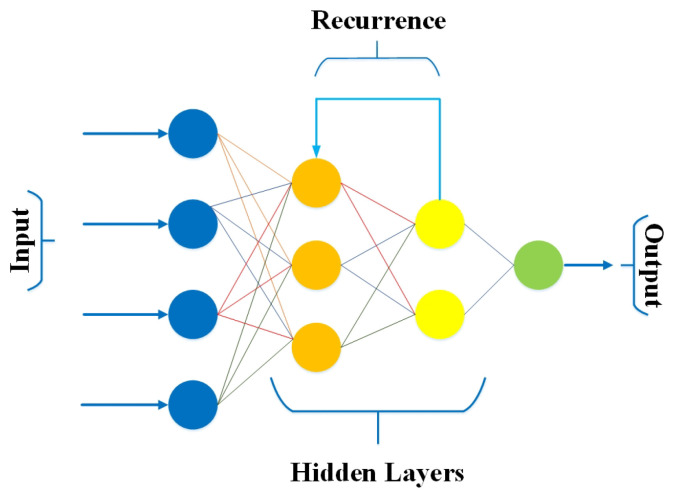
Architecture of RNN.

Therefore, RNNs have promising applications in speech recognition, text summarization, prediction problems, face detection, music composition, and language processing [57]. Figure 6 shows a simple RNN architecture consisting of an input layer, one or more hidden layers, and an output layer.

#### 3.6.1. Long Short-Term Memory (LSTM) Networks

**Figure 7:**
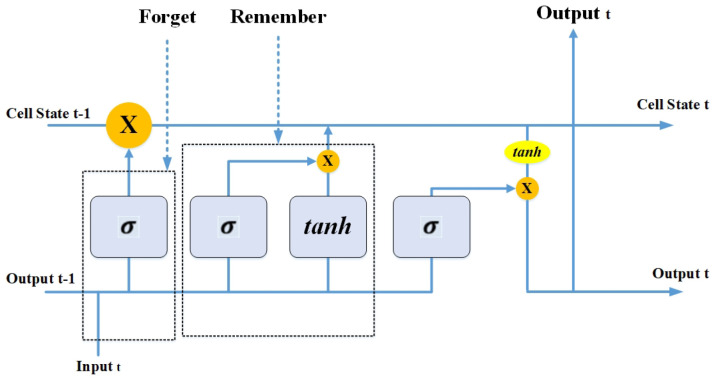
Architecture of LSTM.

LSTM is a type of RNN that comprises a “memory cell” that can preserve information in memory for long periods. A set of gates is used to regulate when information arrives in the memory of a cell. There are three types of gates, i.e., input gate, output gate, and forget gate. The input gate selects how much information from the last example will be reserved in memory; the output gate controls the amount of data delivered to the next layer, and forget gates regulate the tearing rate of memory keeping. This planning lets them learn longer-term reliance [58]. Figure 7 shows the architecture of LSTM consisting of three gates, i.e., forget gate, remember gate, and output gate, deciding the data sequence. In LSTM, the *tanh* activation function controls the values that flow across the network, whereas the sigmoid activation function (σ) is used at the gates as a gating function.

#### 3.6.2. Gated Recurrent Unit (GRU) Networks

The recurrent neural network (RNN) architecture, known as a “gated recurrent unit (GRU)”, is intended to capture long-term dependencies in a sequential input efficiently. It was developed to improve the standard RNN model, solving issues including the vanishing gradient problem. Moreover, LSTM has three gates—input, output, and forget; GRU’s bag only has two gates—reset and update. A GRU may learn to selectively update and preserve information over time by employing the update and reset gates, which enables it to capture long-term dependencies more efficiently than a conventional RNN [59]. GRUs are extensively used in several sequential data tasks, including machine translation, speech recognition, and natural language processing.

### 3.7. Sequence-to-Sequence Models

**Figure 8:**
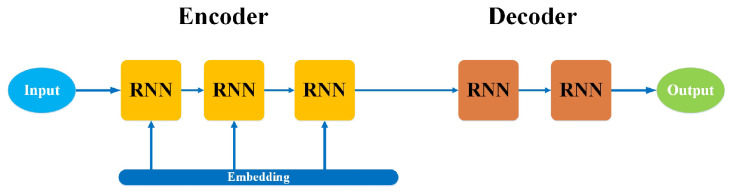
Architecture of sequence-to-sequence model.

A sequence-to-sequence model comprises two RNNs with additional encoder and decoder modules. The encoder is used for the input data, and the decoder delivers the output. The encoder and decoder work in parallel using the same or different parameters [60]. Contrary to the actual RNN, this model only applies when the input data’s length and output data’s size are equal. These models are primarily used in chatbots, machine translation, and question-answering systems, despite having similar help and curbs to the RNN. The layout of the sequence-to-sequence model, consisting of an encoder and a decoder, is shown in Figure 8. An encoder and a decoder are independent essential neural network models integrated into a massive network to create an output representation sequence. The encoder and decoder comprise two RNNs that act as an encoder and a decoder pair. The encoder receives an input of variable length and maps it to a fixed-length vector, then sends it to the decoder that maps the fixed-length vector back to a variable length as a target sequence to produce an output. This process is accomplished by utilizing the RNN network inside the encoder and decoder.

### 3.8. Modular Neural Network

**Figure 9:**
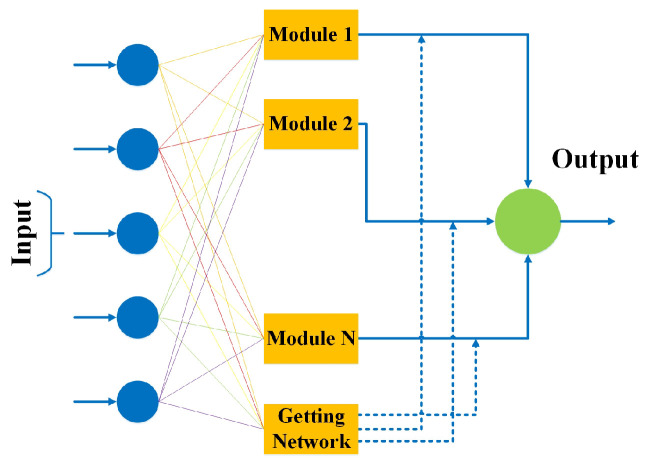
Architecture of modular neural network.

Another type of neural network is a modular neural network consisting of numerous dissimilar networks that can function self-sufficiently to complete various subtasks [61]. During the computation process, the different networks do not cooperate or signal each other to work independently to achieve the output. Thus, a large and complex computational procedure is performed faster by breaking it down into self-governing components. Modular neural networks are efficient and can conduct independent training [62]. Figure 9 shows the architecture of a modular neural network working on the principle of divide and conquer to split significant problems down into smaller manageable parts called modules to produce a single output for each using a module network. All the modules are trained independently, and then the production of each module is stored in a new NN model denoted as getting network in Figure 9 to produce an output.

## 4. CNN Models for COVID-19 Detection

In the previous section, we presented various neural networks. This section highlights the role of CNN in early predicting infectious diseases, especially COVID-19. Since the COVID-19 outbreak in China in December 2019, the global population and economy have been affected badly. To alleviate the spread of COVID-19, timely detection was essential to quarantine infected patients. The primary screening technique used to detect COVID-19 was polymerase chain reaction (PCR) testing; however, it is costly and can fall short of supply [63]. Therefore, an alternative screening method, such as a radiography examination, is used to detect COVID-19, which indicates that people affected with COVID-19 have irregularities in their chests, especially in the lungs. However, specialist radiologists are required to analyze such radiography images, and they might only sometimes be available. Therefore, there is an urgent need for an automated system that can analyze radiography images and lessen the workload of radiologists. In this case, neural-network-based X-ray screening is assumed to be a promising technique to test COVID-19 in asymptomatic patients [64,65]. The leading interest in developing neural-network-based techniques for COVID-19 detection led to the development of many state-of-the-art neural network models to enhance COVID-19 detection accuracy [11,66,67,68]. Therefore, we provide a comprehensive review of existing neural network models to investigate the working of existing models in terms of their achievements and limitations provided in Table 1.

### 4.1. Decompose, Transfer, and Compose (DeTraC)

In [69], the authors present a deep CNN model (DeTraC) that indexes COVID-19 chest X-ray images. DeTraC can compact any abnormalities in the image dataset using a class decomposition mechanism. The DeTraC model consists of three phases. In the first phase, a backbone pre-trained CNN model of DeTraC is trained to fetch the deep local features of every image. In the second phase, a sophisticated gradient descent optimization process is used for training. In the last phase, the class composition layer is utilized to improve the final classification of images. The DeTraC model is trained and tested using a combination of two datasets (80 chest X-ray (CXR) images for regular patients and 116 for COVID-19 patients). The experimental results in [70] show that the DeTraC model exhibits a good accuracy of 93.1% (with a sensitivity of 100%) for identifying COVID-19 X-ray images from normal and severe acute respiratory syndrome cases. However, the small dataset (size) used for training and testing the model degraded its performance in complex scenarios.

### 4.2. COVID-Net

Recently, Want et al. used COVID-Net, a deep CNN model, to classify chest X-ray images into regular and COVID-19-infected patients [71]. To build COVID-Net, the human-driven principled network design prototyping is integrated with machine-driven design consideration to make a personalized network design for recognizing COVID-19 cases from chest radiography images. The authors of [72] used the COVIDx dataset, which comprises 13,975 chest radiography images, to train the proposed COVID-Net model. In [73], a built-in ResNet50 CNN model was first trained and tested on the COVIDx dataset, which achieved a test accuracy of 90.6%. Furthermore, the proposed COVID-Net model was then introduced and tested on the COVIDx dataset, which attained a test accuracy of 93.3%, indicating better performance than ResNet50 on the same dataset. The accuracy of COVID-Net can further be improved by different data augmentation techniques, utilizing network layers, and increasing the dataset size.

### 4.3. Coro-Net

The authors in [74] propose another deep CNN model, Coro-Net, to detect COVID-19 infection from chest X-ray images automatically. The foundation of Coro-Net is on Xception architecture, which is pre-trained on ImageNet and then trained on a combined dataset prepared using two publicly available datasets. The dataset used to train Coro-Net comprises 1300 images with an overall accuracy of 89.6%. Although Coro-Net shows promising results on a small dataset, its efficiency can be increased using a large dataset and minimum data preprocessing.

### 4.4. OptCoNet

The authors in [75] propose an optimized CNN for an automatic diagnosis of COVID-19 (OptCoNet). The OptCoNet building blocks have enhanced feature extraction and classification modules, and it uses a grey wolf optimizer (GWO) algorithm to optimize the hyperparameters to train the CNN layers. OptCoNet is trained using the datasets collected from two open-source repositories, comprising 2700 images with 900 COVID-19-infected patients’ images and 1800 non-COVID-19 images. The results show that OptCoNet has promising results with an average accuracy of 97.78%. Nevertheless, the limited dataset used to train OptCoNet may lead to the inefficiency of the model.

### 4.5. COVID-MTNet

In [76], the authors present a deep learning neural network model called COVID-MTNet that uses multitask deep learning to detect COVID-19 from X-ray images. COVID-MTNet uses multiple models for different tasks, such as the classification model for COVID-19 detection and the segmentation model for region of interest (ROI) detection. Furthermore, the recurrent, residual neural network (RRCNN) model performs the COVID-19 detection task, and the NABLA-N network executes the infected region segmentation from X-ray and CT images. The dataset used to train the model comprises 5216 image samples. COVID-MTNet has shown 87.26% testing accuracy, which indicates good efficiency and reliability. However, data samples may be increased to validate the model’s robustness and accuracy.

### 4.6. CovNet30

Another CNN-based model for automatically diagnosing COVID-19 from chest X-ray images is presented in [77]. During the training, several submodels are obtained from the Visual Geometry Group, composed of 19 layers, 16 convolutional layers, 3 fully connected layers, 5 MaxPooling layers, and 1 softmax layer to build a 30-layered CNN model (CovNet30), and the resulting submodels are arranged together using logistic regression. The CovNet30 model categorizes chest X-ray images into COVID-19, regular, and pneumonia classes and uses the COVID-19 CXR dataset to train the model. Their COVID-19 CXR dataset consists of 2764 chest X-ray images collected from three open-source data repositories, and it has achieved an accuracy of 92.74% for the classification of X-ray images.

### 4.7. COVIDPEN

In [78], the authors propose a COVID-19 detection technique called COVIDPEN, which uses chest X-rays and CT scans. COVIDPEN utilizes a transfer learning technique to identify COVID-19 patients. The dataset used to train the model comprises 746 image samples. COVIDPEN achieved an accuracy of 96% on the chest X-ray image dataset for the detection of COVID-19. Nevertheless, COVIDPEN also shows 96% accuracy only for a small dataset.

### 4.8. PDCOVIDNet

Another CNN model, Parallel-Dilated COVIDNet (PDCOVIDNet), is proposed in [79] for COVID-19 identification from chest X-ray images. PDCOVIDNet is trained on a dataset comprising 2905 chest X-ray images (COVID-19 cases and healthy controls). The proposed model achieved an average accuracy of 96.58%, indicating the high reliability of PDCOVIDNet. However, the dataset used to train and test the model is very small, and PDCOVIDNet will likely exhibit degradation in robustness and efficiency for larger datasets.

### 4.9. U-Net

In [80], a novel deep learning model was trained on chest X-ray images using U-Net architecture to detect COVID-19. The dataset used to evaluate the model comprises 1000 chest X-ray images comprising 552 normal images, while 448 are COVID-19 affected. Moreover, U-Net has achieved an overall accuracy of 94.10%. However, it shows poor performance for larger real-time datasets.

### 4.10. CapsNet

The authors in [81] present a novel ANN called CapsNet for identifying COVID-19 disease using chest X-ray images with capsule networks. Capsule networks preserve objects’ spots and possessions in the image and model their relationships orderly to overcome the pooling layer issues of feature extraction (i.e., missing small features during feature passing to the next layer). Unfortunately, CapsNet also used a small dataset of 1050 images to train the model, and its performance may degrade for a larger dataset.

**Table 1:** CNN models for COVID-19 detection.

Ref.	Model	Dataset	Image Type	Accuracy	Comments
[66]	DeTraC deep convolutional neural network	80 negative 105 positive	Chest X-ray images	93.1%	This model produces a significant performance on chest radiography images. However, dataset samples are too small to make perception for its performance over a large dataset in an ideal condition.
[74]	Coro-Net deep neural network	310 negative 330 positive	Chest X-ray images	90%	The number of samples is not up to the mark to judge the model performance in a real-time environment. Although accuracy seems good, there are many chances of model overfitting in this case.
[75]	OptCoNet optimized convolutional neural network	1800 negative 900 positive	Chest X-ray images	97.78%	This model achieved significant accuracy. However, the dataset is average in size and cannot be considered ideal for complying the model for real-time analysis and set as an example.
[76]	COVID MTNet deep learning model	1341 negative 3875 positive	Chest X-ray and CT images	84.76%	The number of positive samples is relatively higher than the negative, , which may lead to an unusual behaviour model. Moreover, the model achieved an average accuracy, which may have high chances for underfitting of the model.
[79]	PDCOVIDNet parallel-dilated CNN	1341 negative 1564 Positive	Chest X-ray images	96.58%	The model achieved good accuracy on an average size of the dataset.
[80]	U-Net a novel deep learning model	552 negative 448 positive	Chest X-ray images	94.10%	This model acquires high accuracy on a small dataset, which needs to be better for a reliable model, and results may mislead in critical situations.
[81]	CapsNet a novel ANN model	1050 negative 231 positive	Chest X-ray images	97.24%	Again the model achieved higher accuracy on a small dataset. Consequently, model overfitting can be expected.
[82]	COVID-Net deep convolutional neural network	13,604 negative 2972 positive	Chest X-ray images	92.4%	This model seems quite promising with good accuracy results and a significant number of samples. However, a large set of negative samples can influence the results.
[83]	CovNet30 30-layered CNN model	1139 negative 1625 Positive	Chest X-ray images	92.74%	The model achieves good accuracy, but the number of samples is smaller compared with the performance of other state-of-the-art models trained on a large number of samples.
[84]	COVIDPEN pruned efficiently net-based model	180 negative 566 positive	Chest X-ray and CT images	96%	Due to the significantly small dataset size, despite achieving high accuracy, the reliability of the results is compromised.
[85]	EMCNet CNN and ensemble of machine learning classifiers	2300 negative 2300 positive	Chest X-ray images	98.91%	The model performed significantly well on a balanced dataset and produced high-accuracy results. However, increasing the data size may lead to a slight performance decrease.
[86]	Four-layered CNN model for analyzing CT images	3250 negative 5776 positive	Chest X-ray and CT images	97.8%	The model performed significantly well on a relatively large dataset. However, time and space complexity may vary over time, and several training hyperparameters are also high, which may cause the slow performance of a model in the future with more large datasets.
	ConXNet proposed CNN model	10,192 negative 3616 Positive	Chest X-ray images	97.8%	A proposed model in the paper acquires a significantly good accuracy on a relatively large dataset as compared with other models. Some of the models also achieved good results close to the proposed model. However, this model is trained on a fewer number of hyperparameters by using different techniques, such as batch processing and dropout layers, to overcome overfitting of the model as well. Therefore, the results of the model are reliable but can be improved in the future with more samples.

## 5. Proposed ConXNet Model for COVID-19 Detection

In the previous section, we examined various CNN models for COVID-19 detection. We introduce our new ConXNet model, which offers enhanced accuracy and precision. This model has been trained on diverse datasets of chest X-ray images. The following section presents this novel ConXNet model for COVID-19 detection.

### 5.1. Dataset

As mentioned earlier, we have used several datasets to evaluate the performance of the ConXNet model. The dataset comprises 13,808 chest X-ray images with 3616 COVID-19 data and 10,192 average or non-COVID-19 data [87]. Furthermore, to ensure a robust evaluation of our proposed model, we conducted testing using diverse and representative datasets obtained from publicly available sources. These datasets encompass a wide range of authentic clinical scenarios to reflect the challenges encountered in medical imaging. Specifically, we gathered a comprehensive collection of 2473 chest X-ray (CXR) images from the podcast dataset, which provides a rich and diverse set of patient cases. In addition, we incorporated 183 CXR images obtained from a renowned German medical school, further enhancing the applicability of our evaluation. To further expand the scope and diversity of our assessment, we incorporated 559 CXR images sourced from Kaggle, a popular platform for data science competitions. These images were selected to encompass various pathologies and variations encountered in routine clinical practice. Lastly, we augmented our dataset with an additional 400 CXR images obtained from a reliable source on GitHub. This inclusion allows us to address a more comprehensive array of clinical scenarios and strengthen the validity of the evaluation. By leveraging these diverse and authentic datasets [88,89,90,91,92,93,94], we aimed to thoroughly assess the performance and generalizability of our proposed model, ensuring its reliability and applicability.

### 5.2. ConXNet Architecture

**Figure 10:**
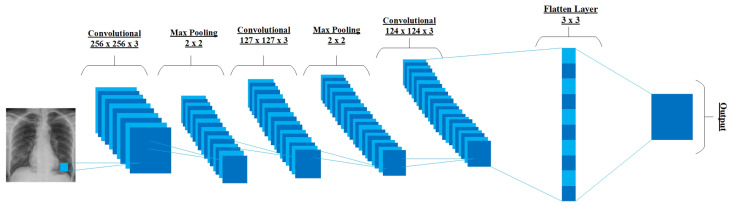
Proposed ConXNet model for COVID-19 detection.

The ConXNet model consists of four blocks, where each block comprises a convolutional layer (Conv), rectified linear unit (ReLU) (operating as an activation function), batch normalization, and MaxPooling layer. Figure 10 represents a detailed architecture of the proposed ConXNet model. X-ray images are fed to the first Conv layer as an input to extract the features (edges, soft edges, blur) from the given input. Once the features are removed, the Conv layer produces a filter matrix called a feature map as an output. After applying different filters on the input image, ReLU is used for nonlinear operations so that the model can learn non-negative linear values. Later on, the rectified feature map is passed through the MaxPooling layer to fetch the most prominent element. Furthermore, batch normalization techniques are applied to prevent the model from overfitting. Once all the convolutional operations are performed, the output is flattened before being sent to the fully connected dense layer to produce the final output. The last, output layer classifies COVID-19 or normal X-ray images from our data.

### 5.3. Experimental Results

**Figure 11:**
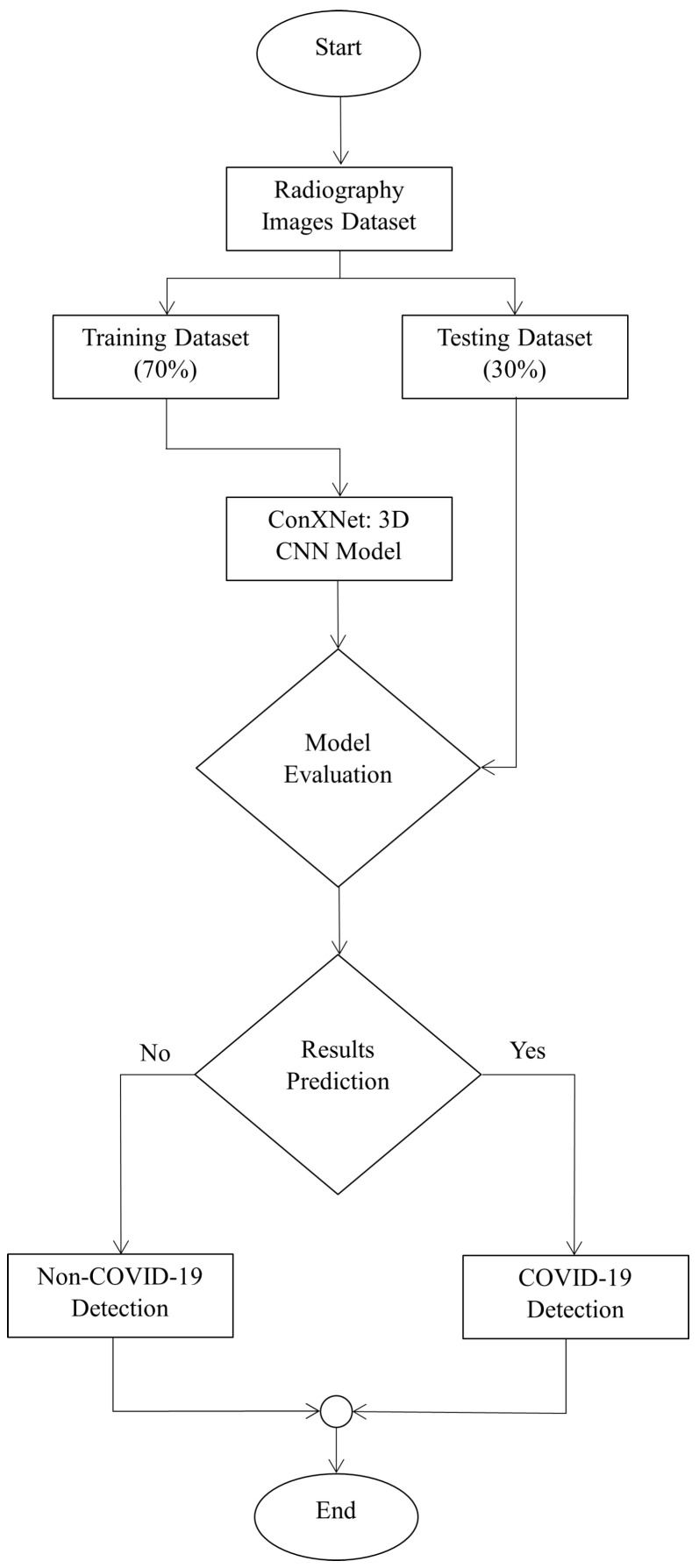
Flowchart of the proposed algorithm.

We have implemented the CNN model in a 64-bit Windows 10 operating system using Python 3.6. The tensor flow framework builds and trains the proposed model using Keras as the back end. The flowchart provided in Figure 11 deliberately explains the overall flow diagram of our proposed model. Before fitting the model, similar images from both classes are selected, such as 3500 for COVID images and 3500 for typical images. A total of 7000 images are used (according to the machine compatibility) for training and evaluation of the proposed method model. Furthermore, the dataset is split into 70% (4900) images for training and 30% (2100) images for testing purposes.

Additionally, the model is compiled using binary cross-entropy (BCE) as a loss function for binary classification. Epochs of 100 and batch sizes 32 are used to fit the model, while 0.001 is used as a preliminary learning rate value. Finally, the Adam optimizer reduces the error rate and automatically tunes the learning rate. It is an efficient variant of gradient descent that prevents the model from hand-tuning the learning rate and does it by itself more quickly and efficiently. The results show that this CNN model achieves an overall accuracy of 97.8%, significantly promising for large datasets. Moreover, we have also evaluated the performance of the proposed model by carrying out a real-world evaluation test. In the following, we discuss the results of our test.

#### 5.3.1. Obtained Results for “COVID-19_Radiography_Dataset”

To effectively observe the performance of the proposed model, the dataset is divided into training and testing with a ratio of 70% and 30%, respectively. The Tensorflow framework builds and trains the proposed model using Keras as the back end. The model is compiled using binary cross-entropy and accuracy performance metrics. Furthermore, learning is first initialized to 0.001 preliminaries to train the model, while 100 epochs are used with 32 batch sizes. Moreover, the error rate is optimized using the Adam optimizer for authentic learning. Finally, three more common performance measures (accuracy, precision, and F1-score) are used to observe the proposed model’s efficiency. It is observed that the model achieved a significant accuracy score of 97.8% with precision and an F1-score of 97.93% and 97.92%, respectively. Table 2 summarizes the results produced by the proposed ConXNet model.

#### 5.3.2. Testing of the Proposed Scheme

**Figure 12:**
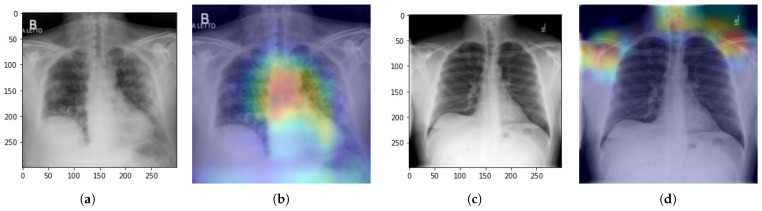
Images used to evaluate the model accuracy with their outputs (**a**). Original image of COVID-19 patient (**b**). Heat map view of COVID-infected region. (**c**) Original image of the normal patient (**d**). Heat map view of the normal image.

After training and testing the proposed model on COVID-19_Radiography_Dataset, we also tested the proposed scheme to evaluate the efficiency and accuracy of the model on unseen data selected from the test dataset. The real-world evaluation experiment gives random images as input to classify whether the images belong to COVID-positive or negative patients. The chest X-ray image is passed to the network as input, and then the image is preprocessed to match the target size input for the proposed model. Moreover, the proposed model predicts the probabilities of the given image, whether it belongs to the COVID class or normal class, with an approximate predicted percentage. The heat map effect is used to visualize the area in the image affected by the disease. These experiments give us a valuable view to evaluate the efficiency of the proposed model in the real world. Figure 12a Illustrates an original X-ray image of a COVID-19 patient, and Figure 12b shows the results after detecting images with heat map highlights of infected regions. Meanwhile, Figure 12c illustrates the original image of the typical patient, and Figure 12d shows after evaluation of the results. To facilitate the reproducibility of our work, and we have made the complete code and trained model available online (https://github.com/azeemchaudhary/CovidXNet/blob/main/Covid_Detection_Model.ipynb (20 May 2023)).

## 6. CNN Models for Detection of Other Diseases

CNN models play a crucial role in Alzheimer’s, cancer, and retinal diseases by analyzing medical images and extracting patterns for accurate diagnosis and treatment. In Alzheimer’s, CNN models analyze brain imaging data to identify abnormalities, track disease progression, and predict outcomes. For cancer, CNN models aid in tumor detection, classification, and staging. In retinal diseases, CNN models diagnose conditions such as diabetic retinopathy and age-related macular degeneration by analyzing retinal images. With their ability to learn complex patterns, CNN models significantly contribute to improving medical imaging analysis, enabling informed decision-making by healthcare professionals and enhancing patient outcomes. This section briefly discusses the role of CNN models in these diseases to highlight their global impact, diagnostic challenges, the complexity of medical images, and research significance, as accurate diagnosis is crucial for these diseases, which profoundly impact health.

### 6.1. Alzheimer’s Disease Detection

Several studies have investigated using CNN to improve Alzheimer’s disease detection and diagnosis. For instance, in [95], the authors propose an automated and reliable deep learning model for detecting and identifying Alzheimer’s disease using magnetic resonance imaging (MRI) and positron emission tomography (PET) that achieved 94.48% average accuracy. In another work [96], an ensemble deep CNN model was developed to detect Alzheimer’s disease using the Open Access Series of Imaging Studies (OASIS) dataset that acquired 93.18% accuracy. In [97], a CNN model developed for Alzheimer’s disease detection uses a brain imaging structured dataset comprising brain MRI images that achieved 96% accuracy, indicating excellent efficiency compared with other state-of-the-art models. In [98], a four-way CNN-based classifier was used that attains 98.8% accuracy by classifying four classes, including Alzheimer’s disease, mild cognitive impairment, late mild cognitive impairment, and no disease using the ADNI dataset. Recently, Alzheimer’s disease has been detected by introducing transfer learning from a dataset of 2D images to 3D CNNs to enable early diagnosis of Alzheimer’s disease using MRI imaging datasets [99].

### 6.2. Cancer Detection

Deep learning methods, such as CNN, can extract hierarchical features from image data without manual selection and are successfully applied in cancer tissue detection. For example, early breast cancer detection, diagnosis, and treatment are possible due to a computer-aided diagnostic (CAD) system based on mammograms analyzed by CNN-based classifiers. In [100], a CAD approach based on in-depth features detected breast cancer using a mammogram image dataset, achieving 81.75% accuracy. In [101], a novel CNN-based model detected lung cancer using computed tomography (CT) scan images and achieved 86.6% accuracy. In [102], a novel CNN-based model is proposed for detecting prostate cancer using the diffusion-weighted magnetic resonance imaging (DWI) dataset, which acquired 84% accuracy. In [103], a fully convolutional network (FCN) was introduced to detect liver cancer using CT scan images, achieving 86% accuracy using a threefold cross-validation technique.

Besides breast cancer detection, CNN has also shown great potential for brain tumor detection, the most frequent and severe type of cancer with a life expectancy of only a few months in the most advanced stages. Therefore, treatment planning for brain tumors is essential in improving patients’ quality of life. In [104], a CNN classification model is proposed for automatic brain tumor detection using brain MRI image datasets. The experimental results show that the proposed model acquired 97.5% accuracy. Another CNN model presented in [105] classifies images for brain tumor detection and achieves 97.87% accuracy. The CNN model presented in [106] performs the classification and segmentation of brain tumors using the MRI image dataset and achieves 94.6% accuracy. In [107], a CNN-based model extracted brain cancers from MRI images with an overall accuracy of 96%. A CNN model for detecting benign tumors was recently introduced in [108] that was trained and tested using BraTS2013 and WBA datasets and delivered 96–99% accuracy.

### 6.3. Retinal Disease Detection

Optical coherence tomography (OCT) imaging makes accurate and reliable diagnoses of retinal disorders, which is critical for clinical relevance. In [109], the authors introduced a novel CNN model for accurate detection and classification into normal, drusen macular degeneration, and diabetic macular edema using an optical coherence tomography (OCT) imaging dataset. Moreover, the Kuan filter is used to input OCT pictures first to reduce intrinsic speckle noise. Furthermore, hyperparameter optimization techniques are used to optimize the CNN network, and K-fold validation is performed to ensure complete utilization of the dataset. As a result, the presented model achieved 95.7% accuracy. Another CNN model presented in [110] accurately detects retinal blood vessels. Recently, in [111], the CNN-based approach localizes, identifies, and quantifies abnormal features in the eye retina using the OCT image dataset and achieves an accuracy of 95.8%. In [112], OCT imaging of the retinas was analyzed using three different CNN models comprising five, seven, and nine layers to identify the various retinal layers, extract usable information, detect new aberrations, and predict several eye abnormalities. Furthermore, the CNN model recently developed in [113] classifies retinal disease using OCT images and achieves 98.73% accuracy. Table 3 summarizes the contributions of the CNN models for other diseases’ detection.

## 7. Discussion, Opportunities, and Open Issues

### 7.1. Discussion

Applying ANNs and deep learning in COVID-19 detection has shown remarkable potential in improving diagnostics and patient care. These models have demonstrated high accuracy and speed in analyzing medical images, allowing for rapid identification of COVID-19 cases. However, it is crucial to acknowledge the limitations and challenges associated with these techniques. The interpretability of deep learning models remains a significant concern, as their decision-making process is often considered a “black box”. Efforts should be focused on developing explainable AI approaches that provide insights into the features and patterns driving the model’s predictions. This would enhance the trust and acceptance of AI-based systems in clinical practice.

Another critical aspect is the ethical use of AI in healthcare. Privacy concerns, data security, and potential biases in AI algorithms must be carefully addressed. Healthcare professionals and AI experts should collaborate to develop guidelines and regulations that ensure patient privacy, informed consent, and appropriate deployment of AI technologies. Transparency and accountability in AI decision-making processes are essential for building public trust and ensuring equitable access to healthcare services. It is also crucial to mitigate biases in training data that could result in COVID-19 and other disease detection and treatment disparities.

Collaboration between interdisciplinary teams is vital to advancing the field of AI in COVID-19 detection. Close cooperation between clinicians, researchers, data scientists, and policymakers is necessary to leverage the potential of AI in a responsible and effective manner. Combining clinical expertise with AI capabilities, we can develop models that improve diagnostic accuracy and provide valuable clinical insights and decision support. Multidisciplinary collaborations can also help address challenges, such as data sharing, standardization of protocols, and validation of AI models across diverse healthcare settings.

### 7.2. Opportunities

The presented review and the proposed model in this paper can serve as a guideline and framework for designing and implementing AI-based COVID-19 and other disease detection systems. It provides a comprehensive overview of the various phases of developing such systems and the key considerations and specifications for each phase. By following the reference model, researchers and developers can ensure a systematic and structured approach to the design and implementation process.

A critical aspect of the reference model is the inclusion of performance measures. These measures provide a quantitative assessment of the effectiveness and reliability of AI-based COVID-19 detection systems. Examples of performance measures may include sensitivity, specificity, accuracy, precision, recall, and F1-score. By defining and evaluating these measures, researchers can assess the performance of their models and compare them with existing approaches, enabling a fair and objective evaluation.

#### 7.2.1. Integration of AI with Internet of Healthcare Things (IoHT)

Integrating AI algorithms with the IoHT, such as wearable sensors and remote monitoring systems, presents real-time data collection and analysis opportunities. By leveraging IoT data; AI models can continuously monitor COVID-19 patients, enabling early detection of deterioration and timely intervention. This integration also opens avenues for personalized medicine and remote patient management, enhancing healthcare accessibility and reducing the burden on healthcare systems. Integrating COVID detection technologies with current technologies, such as the Internet of Healthcare Things (IoHT), presents numerous research opportunities and challenges. One aspect to consider is the tradeoff between learning cost and performance. Deep learning, while achieving good predictive scores, can be computationally expensive. This raises questions about whether opting for weak learners with good features or complex learners with raw data is more beneficial. A potential approach could involve leveraging learned features offline and using weak learners online for real-time decision-making.

Additionally, latency is critical in disease detection systems, as timely data processing is essential for accurate and efficient diagnosis. This calls for investigations into optimizing the tradeoff between learning cost and latency. Techniques such as edge computing can be explored, where data preprocessing and initial feature extraction are performed at the edge devices, reducing the need for transmitting large amounts of data to the cloud. This can help mitigate latency issues and improve real-time decision-making.

In the context of communications, the issue of latency and data processing delays becomes even more relevant. Integrating COVID detection systems with communication networks requires careful consideration of network architecture, protocols, and transmission capabilities. Techniques such as edge caching and distributed processing can be explored to minimize latency and improve the overall system performance.

#### 7.2.2. Federated Learning for Privacy-Preserving Analysis

Federated learning allows collaborative model training on distributed data sources while preserving privacy. This approach can be beneficial in COVID-19, where data privacy is crucial. Researchers can leverage data from multiple institutions without compromising patient privacy by developing federated learning frameworks. This collective analysis can lead to improved models with enhanced generalizability and robustness.

#### 7.2.3. Integration of AI in Telemedicine Platforms

Telemedicine has witnessed significant growth during the COVID-19 pandemic. Integrating AI capabilities into telemedicine platforms can enhance remote diagnostics and triaging. AI models can assist healthcare providers in making accurate and timely decisions based on patient data and medical images. Combining telemedicine and AI can extend healthcare services to remote areas, improving access to quality care and reducing healthcare disparities.

### 7.3. Open Issues

Research, collaboration, and ethical considerations are needed in AI-based COVID-19 detection. Addressing these issues will improve the accuracy and interpretability of AI models and ensure their responsible integration into healthcare systems. By focusing on standardization, interpretability, ethics, and practical integration, we can unlock the full potential of AI in combating the COVID-19 pandemic and future healthcare challenges.

#### 7.3.1. Standardization of Evaluation Metrics and Benchmark Datasets

To enable fair comparisons and promote reproducibility, it is crucial to establish standardized evaluation metrics and benchmark datasets for COVID-19 detection models. Consensus on evaluation protocols, such as sensitivity, specificity, and area under the receiver operating characteristic curve (AUC-ROC), will facilitate meaningful comparisons of different AI models. Additionally, developing diverse and representative benchmark datasets will ensure the generalizability of models across other populations and imaging modalities.

#### 7.3.2. Addressing the Challenges of Model Interpretability and Explainability

Enhancing the interpretability of AI models is vital for gaining the trust of healthcare professionals and end users. Methods for interpreting and explaining deep learning models’ decisions, such as attention mechanisms, saliency mapping, and rule-based explanations, must be further explored. By understanding the reasoning behind AI predictions, clinicians can make more informed decisions and identify potential limitations or biases in the models. Research efforts should focus on developing interpretable AI models that can provide transparent and understandable insights.

#### 7.3.3. Ethical Considerations in AI deployment

The ethical implications of AI in healthcare, including privacy, bias, and transparency, must be addressed. Robust guidelines and regulations should be developed to ensure the responsible use of AI technologies in COVID-19 detection. Ethical frameworks should prioritize patient privacy, informed consent, and equitable access to healthcare resources. Additionally, efforts should be made to address potential biases in training data that could result in disparities in COVID-19 diagnosis and treatment. Discussions and collaborations between healthcare professionals, policymakers, and AI researchers are essential to develop ethical guidelines that align with societal values.

#### 7.3.4. Integration of AI Models into Clinical Workflows

Successful integration of AI models into clinical practice requires addressing practical challenges. User-friendly interfaces, seamless integration with existing healthcare systems, and validation of AI models in diverse clinical settings are crucial steps. Collaboration between AI researchers and healthcare providers is essential to understand the clinical needs and adapt AI technologies accordingly. Moreover, regulatory considerations and compliance with healthcare standards must be considered to ensure AI’s safe and effective use in real-world healthcare environments. Ongoing research and collaboration are needed to overcome these challenges and enable the practical deployment of AI models in clinical workflows.

## 8. Future Research Directions

In this section, we discuss future research directions and challenges, including algorithms complexity, inadequate available data, security and privacy issues, and biosensing integration with ANNs that require considerable attention for enhancing the scope of ANNs for medical treatment and diagnosis.

### 8.1. Complexity

The complexity of neural-network-based disease detection and diagnosis models depends on the computational time and number of samples used to train the network. Since, in medical imagining applications, the accurate detection of disease is more critical, the analysis complexity (such as time and computational complexities) is compromised in most cases [128]. However, in the future, neural-network-based disease detection applications can introduce different methods to address the complex issue of preventing excessive resource consumption without degrading accuracy. For example, 1D and 2D filters can be used at the feature-extraction image-restoration layer with high-resolution images to reduce the complexity [129]. Furthermore, data transformation techniques, including data augmentation [130], variable standardization [131], and proper initialization of ANN [132], can also play a significant role in reducing complexity since these techniques use limited data samples to achieve better results without compromising on accuracy. Moreover, limiting the number of neurons during model training [133], dropout layer technique [134], network pruning techniques [135], normalization techniques, transfer learning techniques, and brute-force exploration approach [136] can also be used to minimize complexity.

### 8.2. Algorithm Selection

Another challenging issue for neural-network-based infectious disease detection is the selection of the proper algorithm. ANN algorithms are generally categorized as supervised learning and unsupervised learning, compromising of various algorithms, including decision trees (DT) [137], naïve Bayes (NB) [138], support vector machine (SVM) [139], random forest (RF) [140], k-nearest neighbour (KNN) [139], and k-means [141] for executing different operations. Moreover, algorithm selection based on the dataset also plays an essential role in achieving the desired results.

### 8.3. Deficient Training Data

In addition to algorithm selection, another major issue is obtaining valuable information from the raw data. The ANNs are primarily applied to large datasets and composite models that need extensive training to obtain the required training effects [142]. However, acquiring adequate training data is challenging due to large data unavailability in numerous domains, such as disease detection using image classification. Thus, the need for more available data for training and evaluating the parameters in the neural networks results in network inefficiency and overfitting. For instance, blood pressure measurements in most of the COVID-19 critical patients are erroneous and unstable, which limits the amount of accurate data.

### 8.4. Privacy and Security

One of the key issues with using ANNs for infectious disease detection is privacy and security concerns. Nevertheless, [143] introduced several methods to attain privacy from the "trusted" authority, attackers, and involved entities. Recently, for COVID-19 location-aware apps, a polling mechanism is used to ensure the privacy of the patient infected with COVID-19 from the noninfected patient [144]. This allows noninfected patients to poll the health authority frequently to check whether they have been in close contact with infected patients. Moreover, privacy in the healthcare domain can also be achieved using private messaging systems [145], private set intersection protocols [146], and memo verification methods using cryptography [147]. However, these protocols are computationally intensive, requiring a tradeoff between security and computational efficiency [148]. Therefore, in the future low computational techniques, such as blockchain [149], cloud computing [150], and deep belief neural networks (DBNN), [151] requires more focus to ensure the required level of privacy and security.

### 8.5. Integration of Biosensing and ANN

The tremendous progress in nanotechnology over the past few years has also advanced the development of biosensors for medical applications [152,153]. Biosensors are analytical tools that can observe cellular activity at the nanoscale and identify specific biological strains at the cellular level [22,154]. To overcome the difficulties of intelligent information identification in a biological medium for contagious disease detection, research communities are concentrating on combining AI cognition techniques, such as metaheuristic algorithms and ANN models with biosensing applications [155,156]. Moreover, ANN-integrated biosensing applications can also considerably increase the accuracy and reliability of disease diagnosis as biosensors can generate time-series data that can be trained and tested employing cutting-edge NNs [157].

## 9. Conclusions

This paper thoroughly reviewed the advances in infectious disease detection and diagnostics by ANNs. The detailed discussions reveal that ANN’s ability to solve complex problems has a huge potential to improve disease detection and diagnostic accuracy, leading to advanced patient care and treatment. Besides reviewing the existing ANN-based disease detection models, we propose a new model, ConXNet, which considerably enhances the detection accuracy of COVID-19 patients with low hyperparameters and lesser time complexity. The extensive testing and training of ConXNet using different available datasets show that ConXNet can detect COVID-19 patients significantly well. Finally, we presented the challenges linked to disease prediction using neural networks and future directions. Because this work is mainly focused on the ANN-based disease detection models, it will be interesting to investigate the potential of ANN for targeted drug delivery and advanced surgical procedure in the future. Moreover, we also suggest testing different evolving techniques, such as transfer learning and hybrid schemes, to enhance detection accuracy and precision in medical diagnosis.

## Figures and Tables

**Table 2:** Performance measures of ConXNet model.

Epochs	Accuracy	Precision	F1-Measure
100	97.8%	97.93%	97.92%

**Table 3:** Summary of the contributions of CNN models for other diseases’ detection.

Diseases	Ref.	Model	Dataset	Accuracy
Alzheimer	[114]	Deep CNN for Alzheimer’s disease detection	Total 615 MRI scan images	94.48%
	[115]	An ensemble of deep CNN	OASIS dataset comprises 416 MRI scan images	93.18%
	[116]	CNN for classification of Alzheimer’s disease	ADNI dataset comprises a total of 1455 MRI scan images	96%
	[117]	A Deep CNN based multiclass classification of Alzheimer’s disease	Dataset comprises of total 355 MRI scans images	98.8%
Cancer	[118]	Breast cancer detection using extreme learning machine based on feature fusion with CNN deep features	Total 400 mammography images	81.75%
	[119]	Lung cancer detection and class- ification with 3D CNN	Kaggle Data Science Bowl (DSB) comprises a total of 1397 MRI scan images	86.6%
	[120]	Prostate cancer detection using deep CNN	Diffusion-weighted magnetic resonance imaging dataset comprises a total of 427 images	84%
	[121]	Fully CNN for liver segmentation and lesions detection	Dataset comprises of total 88 CT scan images	86%
	[122]	Brain tumor classification using CNN	Radiopaedia and brain tumor image segmentation benchmark 2015 datasets are used	97.5%
	[123]	Brain tumor detection using CNN	BRATS dataset comprises 217 MRI images	97.87%
	[124]	Brain tumor classification and segmentation using faster R-CNN	A total of 218 MRI images are used	94.6%
	[125]	Brain tumor classification in magnetic resonance images using deep learning and wavelet transform	MRI image dataset	96%
	[126]	Deriving tumor detection models using CNN from MRI of human brain scans	BraTS2013 dataset and WBA dataset are used	96-99%
Retinal	[127]	Deep CNN framework for retinal disease diagnosis	OCT image dataset containing a total of 12,000 images	95.7%
	[111]	Detection of retinal abnormalities using CNN	Dataset comprises a total of 1110 fundus images	95.8%
	[112]	DL-CNN-based approach diagnosis of retinal diseases	OCT image dataset comprises a total of 84,495 images	96.5%
	[113]	Retinal disease classification using CNN algorithm	OCT image dataset comprises a total of 108,312 images	98.73%

## Data Availability

The data presented in this study are available on request from the corresponding author.

## Abbreviations

The following abbreviations are used in this manuscript:

1D	one (1) dimensional
2D	two (2) dimensional
3D	three (3) dimensional
AI	artificial intelligence
ANN	artificial neural network
CART	classification and regression tree
CNN	convolutional neural network
CT	computed tomography
DBNN	deep belief neural networks
DeTraC	Decompose, Transfer, and Compose
DT	decision tree
FNN	feed-forward neural network
GRU	gated recurrent unit
KNN	k-nearest neighbor
LSTM	long short-term memory
ML	machine learning
MLP	multilayer perceptron
NB	naïve Bayes
NN	neural network
PCR	polymerase chain reaction
PNN	probabilistic neural networks
RBF	radial basis function
ReLU	rectified linear unit
RF	random forest
RRCNN	recurrent, residual neural network
ROI	region of interest
RNN	recurrent neural network
SVM	support vector machine
